# Cognitive deficits and educational loss in children with schistosome infection—A systematic review and meta-analysis

**DOI:** 10.1371/journal.pntd.0005524

**Published:** 2018-01-12

**Authors:** Amara E. Ezeamama, Amaya L. Bustinduy, Allan K. Nkwata, Leonardo Martinez, Noel Pabalan, Michael J. Boivin, Charles H. King

**Affiliations:** 1 Department of Psychiatry, College of Osteopathic Medicine, Michigan State University, East Lansing, Michigan, United States of America; 2 Department of Epidemiology & Biostatistics, University of Georgia, Athens, Georgia, United States of America; 3 Department of Clinical Research, London School of Hygiene and Tropical Medicine, London, United Kingdom; 4 Center for Research and Development, Angeles University Foundation, Angeles City, The Philippines; 5 Center for Global Health and Diseases, PAHO/WHO Collaborating Centre for Research and Training for Schistosomiasis Elimination, Case Western Reserve University School of Medicine, Cleveland, Ohio, United States of America; Ministère de la Santé Publique et de la Lutte contre les Endémies, NIGER

## Abstract

**Background:**

By means of meta-analysis of information from all relevant epidemiologic studies, we examined the hypothesis that *Schistosoma* infection in school-aged children (SAC) is associated with educational loss and cognitive deficits.

**Methodology/Principal findings:**

This review was prospectively registered in the PROSPERO database (CRD42016040052). Medline, Biosis, and Web of Science were searched for studies published before August 2016 that evaluated associations between *Schistosoma* infection and cognitive or educational outcomes. Cognitive function was defined in four domains—learning, memory, reaction time, and innate intelligence. Educational outcome measures were defined as attendance and scholastic achievement. Risk of bias (ROB) was evaluated using the Newcastle-Ottawa quality assessment scale. Standardized mean differences (SMD) and 95% confidence intervals (CI) were calculated to compare cognitive and educational measures for *Schistosoma* infected /not dewormed *vs*. uninfected/dewormed children. Sensitivity analyses by study design, ROB, and sequential exclusion of individual studies were implemented. Thirty studies from 14 countries, including 38,992 SAC between 5–19 years old, were identified. Compared to uninfected children and children dewormed with praziquantel, the presence of *Schistosoma* infection and/or non-dewormed status was associated with deficits in school attendance (SMD = -0.36, 95%CI: -0.60, -0.12), scholastic achievement (SMD = -0.58, 95%CI: -0.96, -0.20), learning (SMD = -0.39, 95%CI: -0.70, -0.09) and memory (SMD = -0.28, 95%CI: -0.52, -0.04) tests. By contrast, *Schistosoma-*infected/non-dewormed and uninfected/dewormed children were similar with respect to performance in tests of reaction time (SMD = -0.06, 95%CI: -0.42, 0.30) and intelligence (SMD = -0.25, 95%CI: -0.57, 0.06). *Schistosoma* infection-associated deficits in educational measures were robust among observational studies, but not among interventional studies. The significance of infection-associated deficits in scholastic achievement was sensitive to ROB. *Schistosoma* infection-related deficits in learning and memory tests were invariant by ROB and study design.

**Conclusion/Significance:**

*Schistosoma* infection/non-treatment was significantly associated with educational, learning, and memory deficits in SAC. Early treatment of children in *Schistosoma-*endemic regions could potentially mitigate these deficits.

**Trial registration:**

ClinicalTrials.gov CRD42016040052

## Author summary

Empirical evidence for cognitive or educational benefits of anti-*Schistosoma* treatment is currently uncertain, despite the recommended practice of wide-scale deworming with praziquantel. We addressed this knowledge gap by synthesizing information from 30 relevant epidemiologic studies reporting on 38,992 children between 5–19 years old from 14 countries. In those studies, *Schistosoma* infection or non-dewormed status was associated with educational loss and cognitive deficits. Specifically, there were small to moderate deficits in both school attendance and scholastic achievement. Similarly, *Schistosoma* infection or non-dewormed status was associated with deficits in learning and memory domains of psychometrically tested cognitive function. However, there was no evidence of *Schistosoma* infection- or non-deworming-associated deficits on tests of innate intelligence or reaction-time. Overall, compared to *Schistosoma-*uninfected or to dewormed children, the presence of *Schistosoma* infection or non-dewormed status was associated with educational, learning, and memory deficits in school-aged children. The combined evidence suggests that early treatment of children in *Schistosoma-*endemic regions could mitigate these deficits.

## Introduction

An estimated 800 million persons in tropical and sub-tropical countries are at risk of infection by one of three main human *Schistosoma* parasites–*S*. *mansoni*, *S*. *haematobium*, and *S*. *japonicum* [1]. As many as 240 million adults and children are actively infected [2–4] resulting in as much as 3.3 million disability-adjusted life years (DALYs) lost per annum due to overt and subclinical morbidities of *Schistosoma* infection [4, 5]. Sub-Saharan Africa is most affected; children from endemic areas are often infected by two years of age and many remain chronically infected throughout their school-age years [6–8]. Periodic mass drug administration (MDA) with praziquantel in school-aged children has been recommended for morbidity control by the World Health Organization [6]. However, *Schistosoma*-infected pre-school children are not routinely treated in such settings, and they constitute a potentially high risk group for accumulation of morbidity [9]. At present, there is no specific guidance for anti-*Schistosoma* drug treatment of preschool children, partly because of the lack of a child-friendly pediatric formulation [10].

Treatment with praziquantel has a demonstrated effectiveness in reducing infection intensity within individuals and in reducing the prevalence of infection within communities [11]. Such treatment results in clear-cut improvements with respect to advanced schistosomiasis-associated morbidities such as urinary tract fibrosis and hepatosplenic disease, including peri-portal fibrosis [12, 13]. Epidemiologic studies have associated *Schistosoma* infections with adverse impacts on anemia, growth [14], fitness [15], pediatric quality-of-life [16], and sub-optimal child development [17]. Definitively linking *Schistosoma* infections to these non-specific and sub-clinical morbidities is complicated in the context of poverty.[8, 12, 17, 18] However, plausible biologic mechanisms of these adverse impacts have been described [2, 19] and the likely underestimation of their morbidity-related health impact has been highlighted [3].

In helminth-endemic regions, the recommendation of periodic deworming of children is explained on the basis of its expected salutary impact on a range of child-health outcomes including anemia [12], nutritional status [14, 17, 18, 20], and overall well-being [16]. In addition, periodic deworming has been linked directly or indirectly to enhancement of school attendance and educational achievement among children enrolled in school [6, 14, 21, 22]. However, the empirical evidence-base for cognitive and educational benefits of deworming remains controversial [23–27]. Recent criticism of the supposed benefits of deworming for educational enhancement has emphasized the undue influence of a single study in evidence reviews [28], which may have led to over-optimistic appraisals regarding the potential health and poverty alleviation benefits of deworming programs [29, 30].

Recent reviews of MDA effects have, thus far, focused on the impact of soil-transmitted helminth infections (STH), but health policy discussions–including those at the World Health Organization [30, 31], have tended to generalize findings to all helminths. Because different parasites can have dramatically different effects in terms of organ-specific and systemic pathologies, it is important now to distinguish the impact and potential benefits of individual anti-helminthic therapies [32]. To date, the evidence base for cognitive or educational benefits of anti-*Schistosoma* treatment has not undergone systematic review. The present systematic review and meta-analysis addresses the following questions: a) among school-aged children examined in the context of cross-sectional or case-control studies, is *Schistosoma* infection associated with worse performance in neurocognitive tests or with educational loss? b) among school-aged children enrolled in prospective studies with specific treatment for *Schistosoma* infection, is lack of treatment with praziquantel associated with worse performance in neurocognitive tests or with educational loss? For our current meta-analysis, we hypothesize that non-treatment or infection with *Schistosoma* infection is associated with educational loss and cognitive deficits in school-aged children from schistosomiasis-endemic regions.

## Methods

### Search strategy

This review, with pertinent information regarding our review protocol, was prospectively registered with the PROSPERO database as follows: http://www.crd.york.ac.uk/PROSPERO/display_record.asp?ID=CRD42016040052 (see Supporting Information file **S1 Text).** We searched Medline, Web of Science, and Biosis electronic databases for original research articles, conference abstracts, or dissertations available as of August 22, 2016. Databases were searched with pre-specified keywords including “bilharzia”, “schistosomiasis”, “*Schistosoma*”, “school attendance”, “attention”, “impairment”, “memory”, “cognition”, among others. The complete search strategy is detailed in **S2 Text**.

### Population, inclusion and exclusion criteria, study design

This systematic review and meta-analysis is focused on school-aged children five years and older. We did not restrict studies according to language, design, or publication date. Both interventional and observational studies were included in this review if they evaluated cognitive function in school-aged children using any psychometric test, or measured school attendance or achievement in relation to infection by *Schistosoma* parasites of any species. We excluded studies exclusively focused on pre-school aged children because of the absence of educational measures and the use of neurocognitive tests (e.g., the Mullen test) that were difficult to classify in terms of neurocognitive domains. We excluded meta-analyses and primary studies of soil-transmitted helminth infections where *Schistosoma* coinfection was absent.

### Comparisons: *Schistosoma* infection vs. no infection & treatment with praziquantel vs. placebo

*Schistosoma* infection status was determined by microscopic examination of stool or urine as appropriate for species. Praziquantel was the primary deworming agent in interventional studies. The primary exposure for this meta-analysis included presence of infection or, operationally, infection was categorically defined based on study design as follows: 1) untreated/placebo *versus* praziquantel-treated in a randomized controlled trial, 2) any, versus no *Schistosoma* infection in cross-sectional studies, or 3) pre-, versus post-praziquantel treatment, or infection-free versus persistent infection, among *Schistosoma*-infected individuals in a longitudinal design study. “Untreated” refers to children determined to be infected but not dewormed.

### Outcomes: Psychometrically assessed cognitive function

In the reviewed studies, psychometrically-assessed cognitive function was measured by a range of instruments that, for the purposes of this meta-analysis, were categorized into four domains: memory, learning/executive function, attention/reaction time, and intelligence (see **S1 Table** for details). The memory domain instruments included tests of working (short-term) memory as well as those of long-term memory. Attention/reaction time tests were those that measured the ability of a child to sustain concentration on a particular object, action, or thought, including their capacity to manage competing demands in their environments. The learning/executive function domain included tests to evaluate children’s performance in goal-oriented behavior, particularly components that are important for scholastic advancement. These test a cluster of cognitive processes that enable children to connect past experience with present action, and by so doing, engage in planning, organizing, strategizing, paying of attention to details, and to emotionally self-regulate, make necessary efforts to remember important details required for attainment of future goals [29]. We included in the ‘intelligence’ domain psychometric tests of intelligence quotient (IQ) that likely measures largely biologically-determined cognitive abilities, in contrast to cognitive performance measures that are environmentally pliable [33].

It was common for studies to use a suite of psychometric instruments to assess a single or multiple cognitive domains in enrolled children. When multiple instruments were used to measure the same cognitive domain, a grand mean of scores and a grand mean of standard deviation (SD) across all instruments were calculated. Thus, for each publication, one overall mean and SD value was determined for each domain. A study could contribute data to different cognitive domains if it used tools spanning across several cognitive domains. However, each instrument only contributed to one single domain of function, as shown in S1 Table.

### Outcomes: Educational loss

Two dimensions of educational loss were tabulated–school attendance and scholastic achievement for children enrolled in school.

*School attendance*: For children enrolled in cross-sectional and longitudinal studies, attendance rate was respectively defined as the number of days children attended school over the past month or over the study period. In case-control studies, the percentage of children enrolled vs. not enrolled in school was calculated for *Schistosoma-*infected and non-infected children.

*School achievement* was assessed across studies based on: i) children’s pass rate on standardized teacher-generated tests; ii) the percent of children who were in appropriate class for age; iii) their enrollment in elite vs. non-elite schools; iv) their scores in the school function domain of pediatric quality-of-life inventory; v) their change in class position after treatment for *Schistosoma* infection; vi) an above average vs. average/below average scholastic performance as rated by a teacher; or vii) their pass rate in any kind of educational test, whether teacher-administered or not.

### Study selection, data extraction and management

Two researchers (LM and AK) independently screened individual articles by title and abstract, after which eighty-eight full text articles were assessed for eligibility for inclusion in this review. Studies were excluded on the following basis: no outcome measure reported (n = 39), non-primary literature or a review article (n = 7), absence of both infection and outcome measures (n = 6), no variation in exposure (n = 2), a limited meeting abstract duplicated by later full publication (n = 3) and a nonhuman study (n = 1) Disagreements between reviewers on inclusion of a given study were resolved by consensus between LM and AK. If no consensus was reached, the article was further evaluated by an additional reviewer (AEE). Thereafter, two researchers (AEE and NP) independently extracted relevant data for meta-analyses. Where differences in approach to standard error (SE) estimation were noted, discrepancy was resolved by consensus between AEE and NP. When a potentially relevant publication did not present needed information for meta-analysis, the authors were contacted to request additional data. If the dataset was publicly available, we obtained needed values directly [28].

### Statistical analysis

The method for deriving SD from respective studies depended on how data were presented in the original reports. Some papers presented median (m) and range (a to b) instead of means and SD. These measures were converted into approximate mean and SD as follows: $\bar{x}\approx \frac{a+2m+b}{4},\;S^{2}\approx \frac{1}{12}\left(\frac{{\left(a-2m+b\right)}^{2}}{4}+{\left(b-a\right)}^{2}\right)$, see [34], where $\bar{x}$ and *S*^2^ refer to the values of mean and variance, respectively. Some studies reported mean of respective measures and 95% confidence intervals. For such studies, SDs were derived as follows: *SD* = *sqrt*(*N*)*(*upper limit*–*lower limit*)/3.92. Other studies presented data on means and their standard errors (SE), and SD was estimated as SD = SE*the square root of N, the study size. For studies presenting data on differences in mean scores between two time points for treated/infected vs. untreated/uninfected groups, appropriate SD for mean difference was calculated using the approach recommended by the Cochrane Collaboration [35].

For the meta-analysis, studies were grouped into six categories of outcomes used to measure cognitive or school-based function: school attendance, school achievement, memory, learning, IQ, or attention. Standardized mean difference (SMD) estimates and 95% confidence intervals (CI) were calculated for each test. SMD estimates were classified as robustly statistically different if their confidence intervals excluded zero. SMD estimates were interpreted based on thresholds described by Cohen [36], as follows: ‘trivial’ (< |0.20|), ‘small’ (≥| 0.20| to < |0.50|), ‘moderate’ (≥ |50| to < |0.80|) or ‘large’ (≥ |0.80|) effects, according to standard practice in social science research.

All analyses and plots were implemented in STATA, versions 11 or 12. Heterogeneity between studies was measured with Higgins’s and Thompson’s I^2^ statistic and chi-square p-values [37]. Where between-study heterogeneity was high, random effects modelling was used to estimate a pooled summary effect across studies [38, 39]. In the absence of heterogeneity, fixed effects modeling was performed [39]. Publication bias was assessed using the Egger test [40]. In sensitivity analysis, we evaluated potential heterogeneity in pooled impact estimates based on: i) observational vs. intervention study design; ii) the quality of original studies based on the Newcastle-Ottawa quality assessment scale; and iii) by *Schistosoma* species. For sensitivity analyses by species, we distinguished between urogenital schistosomiasis (*S*. *haematobium)*, which is often obvious to affected children, and intestinal/hepatosplenic schistosomiasis (*S*. *mansoni/S*. *japonicum)*. The latter two infections are similarly diagnosed by stool exam and infection is seldom obvious to most children. We examined the potential for overly influential publications using the ‘metabias’ function in STATA to evaluate robustness of our pooled estimate, based on sequential removal of individual publications from the calculation of summary estimates. Lastly, we evaluated the impact of year of publication on the stability of the pooled estimate by iteratively including studies based on year of publication–i.e. starting from the earliest to the most recent publication using the ‘metacum’ function in STATA.

### Quality assessment

Our investigation was guided by recommendations of the Preferred Reporting Items for Systematic Reviews and Meta-Analyses (PRISMA) initiative and the Meta-analysis of Observational Studies in Epidemiology (MOOSE) guidelines for observational studies [41]. Quality ranking of each study was implemented using an adapted version of the Newcastle-Ottawa quality assessment scale (NOQAS) [42, 43] to derive a quality score for each investigation with respect to: i) representativeness of the infected population sample or the selection cases and controls (this yielded a score with range 0 to 3*); ii) comparability with respect to known correlates of cognitive function/educational attainment (score range 0 to 6*); iii) the absence of bias in relation to outcome assessment in prospective cohort studies (0 to 3*) or exposure assessment in cross-sectional and case-control studies (0 to 3*). We adapted the comparability segment of this scale to account for confounding effects of age, sex, nutrition, and socioeconomic status in the relation between *Schistosoma* infection and educational/cognitive outcomes. Comparability with respect to these factors was either achieved by design (i.e. age or sex restriction for observational studies, or randomization for RCTs), or analytically, via stratified analyses or multivariable adjustment in regression models. Scores were assigned for attainment of comparability with respect to these factors as follows: age (score = 1*), sex (score = 1*), nutritional status (score = 2*) and socioeconomic status (score = 2*). For each study, the initial raw quality score (max = 12*) was rescaled to match the scale of 9* and then classified as low, high, or very high risk of bias based on precedents in prior literature [42].

## Results

**Fig 1:**
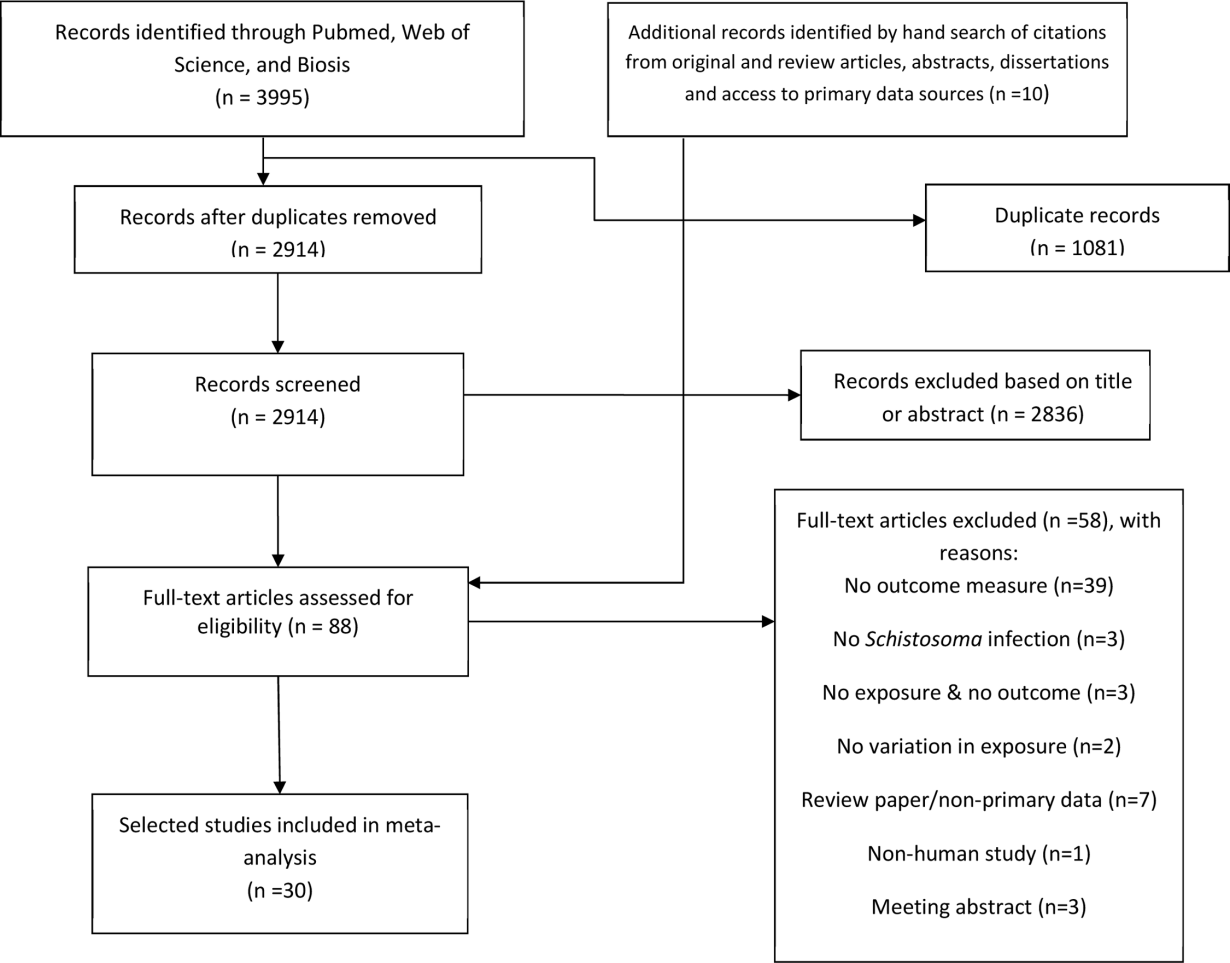
Flow diagram for search and selection of included studies.

Our database search yielded a total 2914 unique records. The screening of titles and abstracts resulted in the exclusion of 2846 records leaving 78 unique papers for full text review. We identified an additional set of ten relevant studies from the bibliographies of relevant articles. Of these 88 articles, 58 were excluded based on full text review for reasons pecified in **Fig 1**.

A total of 30 epidemiologic studies that assessed differences in cognitive test scores (based on psychometric tests) and/or educational status (measured as scholastic achievement or school attendance rate) in relation to *Schistosoma* infection or treatment were selected for inclusion (**Table 1**). Of these, 21 studies were cross-sectional [16, 44–56] or case-control [57–63] design where children were classified based on presence vs. absence of *Schistosoma* infection. Seven were longitudinal studies or pre-post intervention studies that featured screening and treatment for *Schistosoma* infections at the time of first assessment [28, 64–68]. In these studies, outcome contrasts were made according to: a) the duration of persistent infection vs. duration of an infection-free interval, and b) the number of children for whom intensity of infection at last follow-up remained lower vs. the number of children for whom there was no change from baseline infection status after treatment with praziquantel.

**Table 1:** Characteristics of 30 eligible studies of educational or cognitive loss in relation to *Schistosoma* species infection^a^.

Author	Design	Age (years)	N for Infected, Not Treated, or Pre-treatment	N for Uninfected, Treated, or Post-treatment	Outcome Domain(s)	Evaluated by	*Schistosoma* Species Involved	Country
*Loveridge 1948*	CC	11–18	91	108	Achievement	Attendance of elite vs. non-elite school	*S*. *haematobium*	South Africa
*Jordan 1962*	Cohort	12–19	58	58	Achievement	Class Rank Improvement	*S*. *haematobium*	Kenya
*Goldin 1972*	CS	9–12	112	80	Achievement	Teacher ranking of scholastic ability	*S*. *haematobium*	Zambia
*Bell 1973*	Cohort	8–12	69	69	IQ	Ravens Progressive Matrix	*S*. *haematobium & S*. *mansoni*	South Africa
*Castle 1974*	CS	11–18	26	308	Achievement, Learning, Memory	Thurstone Mental Abilities Test	*S*. *haematobium*	South Africa
*Epstein 1974*	CS	13–14	26 to 43	132 to 224	Learning, Achievement, Attendance	Nelson Reading Test, Class Rank, Attendance Rate	*S*. *mansoni*	St. Lucia
*Ejezie 1981*	CS	6–15	164	517	Achievement, Attendance	% Passing in last Year, % Attendance	*S*. *haematobium*	Nigeria
*Haycock 1983*	CS	6–15	481	353	Achievement	% in age-appropriate class	*S*. *haematobium*	South Africa
*el-Hawy 1990*	CS	13–17	300	300	Achievement, Attendance	Pass rate, Attendance	*S*. *haematobium S*. *mansoni*	Egypt
*Kimura 1992*	Cohort	9–19	49	49	Attention	Tanaka Binet Intelligence Test	*S*. *haematobium*	Kenya
*Ekanem 1994*	CS	5–15	177	285	Achievement, Attendance	Teacher Given Test, % attendance	*S*. *haematobium*	Nigeria
*Hussein 1996*	CS	6–18	6471 (Upper) 11080 (Lower)	5149 (Upper), 3006 (Lower)	Enrollment rate	% Attendance	*S*. *haematobium*	Egypt (Upper & Lower regions)
*Fentiman 1997*	CC	6–18	130	239	Attendance	% enrolled in school	*S*. *haematobium*	Ghana
*de Clerq 1998*	CS	6–11	203	263	Attendance	% Attendance	*S*. *haematobium*	Mali
*Nazel 1999*	CC	9–12	80	40	Memory, IQ, Attendance, Achievement	VF, WISC, %Attendance, Standardized Test	*S*. *mansoni*	Egypt
*Nokes 1999*	RCT	5–16	89	92	Memory, Attention	Fluency, FR, DSF, CB, Picture Search	*S*. *japonicum*	China
*Useh 1999*	CC	6–12	243	254	Attendance	% enrolled in school	*S*. *haematobium*	Nigeria
*Beasley 2000*	CC	7–12	167	274	Attendance	% Attendance	*S*. *haematobium*	Tanzania
*Meremikwu 2000*	Cohort	8–9	210	203	Achievement, Attendance	Teacher Administered Test, % attendance	*S*. *haematobium*	Nigeria
*Tiruneh 2001*	CC	6–15	597	518	Enrollment rate	% Attendance	*S*. *mansoni*	Ethiopia
*Jukes 2002*	CS	9–15	241	97	Memory, Learning, Reaction Time, Achievement	DS, WF, CB, SWLT, Stroop, CRT, PBT, SS, Reading, Spelling, Math	*S*. *haematobium +/-* Hookworms	Tanzania
*Miguel 2004*	Cohort	6–18	92 Persistently Infected	407 Uninfected both years	Enrollment rate, Achievement	% Attendance, Change Scores over 2 years	*S*. *mansoni*	Kenya
*Ezeamama 2005*	CS	6–18	244	75	Memory, Learning, IQ	VF/WRAML Memory, WRAML Learning, PNIT	*S*. *japonicum*	The Philippines
*Mekheimar 2005*	CC	6–12	57	42	Attendance	% enrolled in school	*S*. *mansoni*	Egypt
*Grigorenko 2006*	RCT	11–13	92 Screened Not Treated	74 Screened & Treated	Memory, Learning, Reaction Time, Achievement	DS, WF, CB	*S*. *haematobium +* hookworms	Tanzania, Africa
*Berhe 2009*	CS	5–18	219	114	Attention	% symptoms leading to distraction in class	*S*. *mansoni*	Ethiopia
*Ezeamama 2012*	Cohort	6–18	214 Not Cured/re-infected	39 Not re-infected	Memory, Learning, IQ	VF/WRAML Memory, WRAML Learning, PNIT	*S*. *japonicum*	The Philippines
*Terer 2013*	CS	5–18	352	450	Achievement	Score in school functioning	*S*. *haematobium*	Kenya
*Hurlimann 2014*	Cohort	5–14	130	89	Memory, Attention	Digit Span Test, Code Transmission Test	*S*. *mansoni +* STH	Côte D’Ivoire
*Rasoamanamihaja 2016*	CS	7–10	684	1274	Enrollment rate	% regular vs. non-regular attendance	*S*. *haematobium + S*. *mansoni*	Madagascar

^a^Abbreviations: CC, Case-Control; CS, Cross-sectional; RCT, Randomized controlled trial; VF, verbal fluency; WRAML, wide range assessment of memory and learning; PNIT, Philippine non-verbal intelligence test; DS, Digit Span; WF, word fluency; CB, Corsi Block; SWLT, Spanish word learning task; FR, Free recall; DSF, Digit Span Forwards; CRT, choice reaction time; PBT, peg board task (dominant & non-dominant hand); SS = silly sentencies.

Two studies were randomized controlled studies. Only one included study was a classic placbo-controlled randomized-trial intervention in which children with *Schistosoma* infection were randomized to praziquantel vs. placebo/no treatment [69]. The other study randomized children to screening vs. non-screening for *Schistosoma* infections [70]. Children in the non-screened arm remained untreated (although that sample was subsequently tested for infection to distinguish infected from uninfected children). In that study, among children randomized to screening, those found to be *Schistosoma*-infected were given treatment, and we were thus able to derive differences in cognitive test scores between *Schistosoma-*infected/treated vs. *Schistosoma-*infected/not-treated children (**Table 2**). The median follow-up duration across the nine logitudinal studies was 12 months. Minimum follow-up duration was one month and maximum follow-up duration was 36 months. In four of the longitudinal studies, follow-up duration was 6 months or less. In another four studies, follow-up duration was more than 12 months. One study had a 12 month follow-up.

**Table 2:** Pooled estimates of *Schistosoma* infection/non-treatment effects on educational/cognitive loss–Evaluation of heterogeneity and publication bias.

Cognitive Domain	# Studies	SMD (95%CI)^†^		Heterogeneity test		Publication bias	Studies Included
				P-value^φ^	*I*^2^ (%)	P-value^α^	
Memory	8	-0.28 (-0.52, -0.04)		0.0001	78.6	0.786	[42, 48, 52, 58, 62, 67, 76]
Learning	6	-0.39 (-0.70, -0.09)		0.0001	79.4	0.793	[42, 47, 48, 52, 67, 76]
Intelligence Quotient Based Assessments	4	-0.25 (-0.57, 0.06)		0.008	74.8	0.450	[48, 58, 76, 77]
Reaction Time	6	-0.06 (-0.42, 0.30)		0.030	88.5	0.142	[41, 52, 62, 64, 66, 67]
**Educational Loss Assessments**							
Achievement	16	-0.58 (-0.96, -0.20)	0.0001		97.9	0.595	[15, 25, 42–47, 49, 50, 52, 56, 58, 63, 65, 67]
School Attendance	16	-0.36 (-0.60, -0.12)	<0.001		98.7	0.991	[15, 25, 43–47, 49–52, 54–60, 63, 65, 67]

^†^ SMD < 0 suggests a negative effect of infection/non-treatment on the indicated outcome; SMD > 0 indicates a positive effect of infection on respective outcomes.

φ: measures the extent to which there is heterogeneity across studies in terms of underlying results.

α: evaluates the tendency for increased publication of studies that show a statistically robust finding; here, a P < 0.05 suggests presence of publication bias.

In all, a total of 38,992 children between the ages of 5 to 19 years from 14 countries in three continents–Africa, Asia and North America, were included in this review. The vast majority of studies were of children from Africa–Nigeria (n = 4), Egypt (n = 4), South Africa (n = 4), Tanzania (n = 3), Kenya (n = 4), Mali (n = 1), Côte D’Ivoire (n = 1), Zambia (n = 1); Ghana (n = 1) and Ethiopia (n = 1). Three studies were conducted in Southeast Asia or Asia [The Philippines (n = 2) and China (n = 1)], one study was conducted in St. Lucia (Caribbean), and another study was implemented in Madagascar.

A total of 36,626 children were studied in the context of cross-sectional or case control studies. Of these, cognitive test scores and/or indicators of educational loss were measured in 23,126 (59.3%) children infected with one of three schistosomiasis species (*S*. *haematobium* (20 studies), *S*. *japonicum* (3 studies), or *S*. *mansoni* (10 studies)). These infected children were compared to 13,835 (40.6%) children without *Schistosoma* infection. A total of 2,366 children were studied in the context of randomized intervention studies including praziquantel vs. placebo, or prospective treatment studies that included baseline and follow-up assessments of cognitive function (**Table 2**). Of the 30 studies included in this review, three (10%) and 16 (53.3%) were judged to be at very high or high risk of bias, respectively, per the NOQAS (**Table 3**).

**Table 3:** Quality of evidence from individual studies included in the meta-analysis^a^.

			Newcastle Ottawa Quality Assessment Scale				
Study ID	Design	Description	Selection max = 3*	Comparability max = 6*	Exposure /Outcome max = 3*	Scaled Quality Score max = 9	Risk of Bias
**Grigorenko 2006**	RCT	Randomized 254 Tanzanian children 11–13 years old to screening vs. no screening with 16 months of FU. Screened, infected children were treated with ALB + PZQ. All randomized to no screening were untreated but study distinguished "infected not treated" from uninfected/untreated. Analyzed for association between infected/untreated status, infected/treated vs. uninfected/not treated status on cognitive function with statistical control for multiple confounders.	***	******	***	9	Low
**Ezeamama 2012**	Cohort	Treatment reinfection study of 253 schisto infected Filipino children 6–18 years old followed for 18 months with repeated assessment for infection and cognitive function. Evaluated association between infection free duration and performance in four cognitive tests. Controlled for: age, sex, nutritional status, socioeconomic status, coincident STH, and other factors.	***	******	***	9	Low
**Nazel 1999**	CC	120 Egyptian children 9–12 years old. Infected cases (mild & moderate/high intensity, n = 80) matched to uninfected classmate controls (n = 40). Analyses controlled for age. Data on socioeconomic status of both parents, nutritional status, crowding index, and number of siblings did not differ by infection category.	***	******	**	8	Low
**Nokes 199967**	RCT	Placebo controlled 2x2 intervention trial among 181 Chinese children 5–16 years old with allocation to treatment with: PZQ with ALB-placebo, ALB with PZQ-placebo, PZQ and ALB, or PZQ-placebo and ALB-placebo; FU duration = 3 months.	***	******	**	8	Low
**Jukes2002**	CS	338 Tanzanian children 9–15 years old. Included uninfected, moderate, or heavy schistosome infection, with or without coinfection with moderate intensity hookworm. Multivariate control for multiple confounders including SES, nutritional indices, inflammation, and malaria coinfections.	***	******	**	8	Low
**Miguel 2004**	Cohort	Prospective investigation of scholastic achievement and attendance by infection status over 12 months in 499 Kenyan children 6–18 years old. Sample restricted to those present at both FU periods. Robust control for confounding covariates.	***	******	**	8	Low
**Ezeamama 2005**	CS	319 children 6–18 years Filipino children. Controlled for age, sex, hemoglobin status, nutritional status, socio-economic status, and coincident STH infections	***	******	**	8	Low
**Berhe 2009**	CS	Included 333 Ethiopian children 5–18 years old. Multivariable investigation of infection-related differences in psychometric tests. Controlled analytically for several confounders including SES, nutritional status. The surrogate for attention "severe cramps distracting class attentiveness" is inherently subjective.	**	******	**	8	Low
**Terer 2013**	CS	Compared school functioning for schistosome-infected and uninfected Kenyan children 5–18 years old. Controlled for age, sex, nutritional, and socioeconomic confounders via multivariable analyses.	***	******	**	8	Low
**Hurlimann 2014**	Cohort	219 Ivorian children 5–14 years old. Repeated treatment for schistosome and STH infection with 5 months follow-up. Controlled for age, sex, socioeconomic, and nutritional status	***	******	*	8	Low
**Epstein 1974**	CS	Enrolled 267 St. Lucian children 13–14 years old. Compared outcomes for children with infection and uninfected. Age, sex, and SES adjusted for in multivariable analysis.	***	****	**	7	Low
**Bell 1973**	Cohort	138 South African Children 8–12 years old. Analyses compared infected and uninfected children with respect to change in IQ test over 12 months of repeat testing. Authors state that each child was paired (pairing factors unspecified) to eliminate variation due to age, sex, grade, and school. Mostly descriptive analyses reported.	***	***	**	6	High
**Fentiman 1997**	CC	Enrolled 352 Ghanaian children 6–18 years old. Compared schistosome infection in enrolled and unenrolled school aged children matched for age and sex or class (if age-inappropriate for class). No evidence of multivariable analysis but confounding by age, sex, and to some extent SES, is addressed by matching factors.	***	***	**	6	High
**Jordan 1962**	Cohort	Enrolled 116 boys 12–19 years old from hyper endemic area around Lake Victoria. Treated with Lucanthone hydrochloride or TwSb; untreated boys served as controls. Scholastic ability was assessed at enrollment and 6 months later. Boys in treatment group improved their class position over six months vs. those uninfected and/or infected but not treated.	***	*	***	5	High
**Kimura 1992**	Cohort	Included 49 Kenyan children 9–19 years old confirmed to be schisto infected. Allocated to PZQ or no treatment without randomization. There was matching by grade level and pre- vs. post-enrollment assessment of cognition over 1 month. No difference in groups by age, sex, infection intensity, or scores at enrollment. Scores improved for treated but not for untreated children. No evidence of control for sex, SES, or nutritional status in multivariable analysis.	***	*	**	5	High
**Ekanem 1994**	CS	462 infected and uninfected Nigerian children 5–15 years old. Infected children were matched to uninfected children by age and sex. No multivariable analyses; no control for socioeconomic or nutritional status.	***	**	**	5	High
**de Clerq 1998**	CS	Study of 466 infected & uninfected Malian children, 6–11 years old. Association age- and sex-adjusted in multivariable analysis.	***	**	**	5	High
**Meremikwu 2000**	Cohort	210 schistosome infected Nigerian children, all 8–9 years old, were treated with PZQ, followed for 36 months, and screened yearly for reinfection. Re-infected children were retreated. Retention was high. Did not control for sex, SES, or malnutrition.	***	*	***	5	High
**Rasoamana-mihaja 2016**	CS	Enrolled 1958 children 7 to 10 years old from Madagascar as part of a cluster randomized study including 29 sentinel sites. 20 school attending and 4 non-school attending children from each cluster were randomly selected analyzed for relationship of infection (prevalence and intensity) to school attendance. No difference between attendees vs. non-attendees with respect to infection. However, non-attendees had higher intensity of infection.	***	**	**	5	High
**Castle 1974**	CS	Included 334 South African children 11–18 years old with or without subclinical schistosome infection. Measured data on father occupation as SES surrogate, behavioral risk factors, and pupil factual knowledge of infection cause and prevention, but no evidence of multivariable analysis.	***		**	4	High
**Ejezie 1981**	CS	Included 681 Nigerian children 6–15 years old. Descriptive analyses of educational loss by infection status. No evidence of adjustment for SES, age, sex, or nutrition.	***		**	4	High
**el-Hawy 1990**	CS	Enrolled 600 Egyptian boys 13–17 years old. Descriptive analysis of school performance by schistosome infection status. No control for age, SES, or nutritional status.	***		**	4	High
**Hussein 1996**	CS	Comparison of infection prevalence among enrolled and unenrolled school children in upper Egypt (n = 11,620) and lower Egypt (n = 14,806). Infection prevalence and cultural practice with respect to education of children differ by Northern vs. Southern Egypt. All analyses region-stratified, hence we maintain Upper and Lower Egypt as distinct regions contributing unique data points in this meta-analysis. Children were 6–18 years old.	***		**	4	High
**Useh 1999**	CC	Enrolled 560 Nigerian children 6–12 years old. School attendance rate and non-enrollment rate were determined based on head of household recall for index child. Potential misclassification of enrollment due to recall bias.	*	**	**	4	High
**Beasley 2000**	CC	Enrolled 441 Tanzanian children 7–12 years old. Comparison of enrollment rate in infected and uninfected children. Mostly descriptive analysis presented but information on SES, nutritional status and other factors evaluated by infection status.	***		**	4	High
**Tiruneh 2001**	CC	Enrolled 1,115 Ethiopian children, 6–15 years old. Comparison of schistosome infection prevalence among enrolled and non-enrolled children. Descriptive analysis with high potential for residual confounding by SES, nutritional status, etc.	***		**	4	High
**Mekheimar 2005**	CC	Enrolled 99 Egyptian children 6–12 years old. Comparison of enrollment rate by infection status via descriptive analyses.	***		**	4	High
**Loveridge 1948**	CC	Included 199 South African children, 11–18 years old. Descriptive comparison of enrollment in elite vs. non-elite school by schistosome infection status.	**		**	3	Very high
**Goldin 1972**	CS	Enrolled 192 Zambian children 9–12 years old. Descriptive analysis of subjective teacher ranking of index student as above or below average scholastic achievement by schistosome infection status.	**		**	3	Very high
**Haycock 1983**	CS	834 South African children 6–15 years old. Measured whether students were at age appropriate classes or not by schistosome infection status. No information on confounders beyond age. No multivariable analysis.	**		**	3	Very high

^a^Abbreviations: CC, Case-Control; CS, Cross-sectional; RCT, Randomized controlled trial; FU, Follow up; ALB, albendazole; PZQ, praziquantel; STH, soil-transmitted helminths; SES, socioeconomic standing; TwSb, Stibophen

### *Schistosoma* infection and its associations with school attendance and educational attainment

Sixteen studies evaluated *Schistosoma* infection-related differences in school attendance (**Table 2**). *Schistosoma* infection-associated attendance deficits varied in magnitude and direction by study design. Specifically, we did not find any evidence of association between *Schistosoma* infection and school attendance among the two interventional studies (SMD = 0.03, 95%CI: -0.73, 0.78; **Table 4**); however, the observed infection-associated deficit in school-attendance was robust for the pooled estimate of 14 observational studies (SMD = -0.42, 95%CI: -0.70, -0.14). Compared to uninfected or praziquantel-treated children, the magnitude and direction of infection-associated deficit in school attendance was similar for children infected with *S*. *haematobium* or *S*. *mansoni*. Within strata of study quality, the association between infection and scholastic achievement was directionally consistent and statistically robust (**Table 4**). Across all studies, regardless of design or ROB, a deficit in school-attendance was evident for *Schistosoma*-infected or non-praziquantel-treated children compared to uninfected or praziquantel treated children (n = 15 studies; SMD = -0.36, 95% CI: -0.60, -0.12).

**Table 4:** Pooled estimate of *Schistosoma* infection or non-treatment on educational and cognitive loss in school-aged children from schistosomiasis-endemic regions: Stratified by study design, *Schistosoma* species, and study quality^a^^,^^b^.

		Test of Association			Test of Heterogeneity within stratum		
STRATUM	K	SMD	95% CI	P_A_	P_B_	I^2^	AM
**Memory**							
Interventional Design	4	-0.36	[-0.81, 0.09]	0.12	<0.001	90	R
Observational Design	4	-0.17	[-0.33, 0.01]	0.058	0.61	61	F
*S*. *haematobium*	3	-0.45	[-1.07, 0.17]	0.15	<0.001	88	R
*S*. *mansoni/japonicum*	5	-0.19	[-0.41, 0.04]	0.10	0.02	65	R
Low ROB	7	**-0.27**	**[-0.53, -0.004]**	<0.001	<0.001	81.5	R
All Studies Included	8	**-0.28**	**[-0.52, -0.04]**	<0.001	<0.001	81	R
**Learning**							
Interventional Design	2	**-0.79**	**[-1.19, -0.39]**	<0.001	0.062	71	F
Observational Design	4	**-0.18**	**[-0.34, -0.01]**	0.04	0.576	0	F
*S*. *haematobium*	3	-0.46	[-1.15, 0.23]	0.19	<0.001	90	R
*S*. *mansoni /japonicum*	3	**-0.36**	**[-0.54, -0.18]**	< 0.001	0.14	49	F
Low ROB	5	**-0.41**	**[-0.75, -0.06]**	<0.001	<0.001	83	R
All Studies Included	6	**-0.4**	**[-0.70, -0.09]**	0.001	0.05	79	R
**Reaction time**							
Interventional Design	4	0.13	[-0.03, 0.29]	0.12	0	58.2	F
Observational Design	2	-0.39	[-1.19, 0.40]	0.25	<0.001	84	R
*S*. *haematobium*	3	0.11	[-0.07, 0.29]	0.24	0.38	0	F
*S*. *mansoni/ japonicum*	3	-0.20	[-0.82, 0.42]	0.53	<0.00001	93	R
Low ROB	5	-0.07	[-0.49, 0.35]	0.26	<0.001	89	R
All Studies Included	6	-0.06	[-0.42, 0.30]	0.30	<0.001	88.5	R
**Intelligence**							
All	4	-0.25	[-0.57, 0.06]	0.11	0.008	74	R
Low ROB	3	-0.29	[-0.73, 0.15]	0.19	0.003	84	R
**Achievement**							
Interventional Design	4	-0.35	[-0.71, 0.01]	0.06	<0.001	85	R
Observational Design	12	**-0.65**	**[-1.12, -0.17]**	<0.001	<0.001	98.3	R
*S*. *haematobium*	12	**-0.62**	**[-1.09, -0.14]**	0.01	< 0.001	98	R
*S*. *mansoni*	3	**-0.22**	**[-0.40, -0.05]**	0.01	0.32	11	F
Low ROB	6	-0.08	[-0.21, 0.02]	0.114	0.216	29	F
High ROB	7	**-0.84**	**[-1.52, -0.16]**	<0.001	<0.001	95	R
Very High ROB	3	-0.92	[-2.1, 0.28]	0.185	<0.001	98.5	R
All Studies Included	16	**-0.58**	**[-0.95, -0.20]**	<0.001	<0.001	98	R
**Attendance**							
Interventional Design	2	0.03	[-0.73, 0.78]	0.277	<0.001	96	R
Observational Design	14	**-0.36**	**[-0.64, -0.08]**	<0.001	<0.001	99	R
*S*. *haematobium*	10	-0.29	[-0.59, 0.01]	0.06	< 0.001	99	R
*S*. *mansoni*	4	**-0.26**	**[-0.42, -0.01]**	0.001	0.28	22	F
Low ROB	3	**-0.24**	**[-0.42, -0.07]**	0.006	0.286	20	F
High ROB	13	**-0.34**	**[-0.63, -0.05]**	<0.001	<0.001	99	R
All Studies Included	16	**-0.31**	**[-0.57, -0.05]**	<0.001	<0.001	98	R

^a^Abbreviations: K, number of studies; SMD, standard mean difference; CI, confidence interval; P_A_, P value for association; P_B_, P value for heterogeneity; AM, analysis model: R, Random-effects; F, Fixed-effects; I^2^, measure of variability expressed in %

^b^Values in bold indicate significant associations. SMD < 0 suggests a negative effect of infection/non-treatment on the indicated outcome; SMD > 0 indicates a positive effect of infection on the respective outcome.

The overall finding of schistosomiasis-associated achievement deficit (n = 16 studies; SMD = -0.58, 95%CI: -0.96, -0.20) was directionally consistent but varied in magnitude by study design and study quality **(Tables 3 and 4)**. Specifically, achievement deficit with *Schistosoma* infection was noted in the pooled estimate derived from four intervention studies, but the magnitude of this association was lower and not statistically robust (SMD = -0.35, 95%CI: -0.71, 0.01; **Table 4**). Compared to uninfected or PZQ-treated children, the infection or non-treatment associated deficit in scholastic attainment was directionally consistent across different *Schistosoma* species, although the magnitude of effect was higher for infection with *S*. *haematobium* (SMD = -0.62; 95% CI:-1.09, -0.14) than for infection with *S*. *mansoni* (SMD = -0.22; 95% CI:-0.40, -0.05). Among observational study designs, scholastic achievement deficit was statistically robust and of larger magnitude (n = 12 studies; SMD = -0.65, 95% CI: -1.12, -0.17, **Table 4**). Similarly, *Schistosoma* infection was not associated with deficits in scholastic achievement among studies identified as low risk of bias (n = 6 studies; SMD = -0.08, 95%CI: -0.21, 0.02; **Table 4**). The estimates of infection-related deficit in scholastic achievement increased when study quality was lower: for studies with high risk of bias (n = 7) the SMD = -0.84 (95% CI:-1.52, -0.16); for studies with very high risk of bias (n = 7) SMD = -0.92 95% CI: -2.1, 0.28) although the estimated difference was statistically imprecise (i.e., with a wider CI) among the studies with greatest risk of bias.

### Impact of *Schistosoma* infection on psychometrically evaluated cognitive domains

We found *Schistosoma* infection-associated deficits in memory tests (n = 8 studies; SMD = -0.28, 95% CI: -0.52, -0.04; Table 2). Similarly, *Schistosoma* infection was associated with small-to-moderate deficits in learning tests (n = 6 studies; SMD = -0.39, 95%CI: -0.70, -0.09; Table 2). The *Schistosoma* infection-related deficits in memory tests were directionally consistent by study design; although separate pooled estimates for interventional studies (n = 4 studies; SMD = -0.36, 95% CI: -0.61, 0.09) and observational studies (n = 4 studies; SMD = -0.15, 95% CI: -0.34, 0.03) were not statistically robust. For *Schistosoma* infection-related association with learning, pooled estimates suggested the presence of deficits for infected/non-dewormed vs. uninfected/praziquantel-treated children (n = 6 studies; SMD = -0.39, 95% CI: -0.70, -0.09). However, the magnitude of this association differed according to study design. Here, larger pooled standardized differences were realized for interventional studies (n = 2 studies; SMD = -0.79, 95% CI:-1.19, -0.39) than for observational studies (n = 4 studies; SMD = -0.18, 95% CI: -0.35, -0.01). *Schistosoma* infection was not significantly associated with performance on tests of reaction time (n = 6 studies; SMD = -0.06, 95% CI: -0.42, 0.30) or performance in tests of innate intelligence (n = 4 studies; SMD = -0.25, 95% CI: -0.57, 0.06). For all psychometrically-assessed cognitive domains, the overall findings were not sensitive to study quality. Of note, the majority of publications with psychometrically-evaluated endpoints had low risk of bias and produced pooled estimates similar in magnitude and direction to the overall results (**Table 4**).

### Sensitivity analyses

High levels of heterogeneity were observed across included studies (P ≤ 0.03, I^2^ ≥ 74.8%; **Tables 2 and 4**) for all outcome measures, but there was no evidence of undue influence by individual studies or of publication bias among the included studies (Egger’s test, all P-value ≥ 0.142, Table 2). Overall, inferences based on pooled estimates were generally insensitive to differences in study design (**Table 4**), to the exclusion of individual studies (**S2 Table**), or to the influence of publication year **(S1 Fig**).

## Discussion

### Principal findings and interpretations

This systematic review and meta-analysis of the cognitive and educational impact of *Schistosoma* infection in school-aged children supports the hypothesis that infection is associated with reduced school-attendance, with deficits in scholastic achievement and deficits in memory and learning domains of psychometrically evaluated cognitive function. It has previously been conjectured that *Schistosoma* infection may affect school attendance, scholastic achievement, and cognitive function, either directly through via deposition of *Schistosoma* eggs within the central nervous system, via physical discomfort and subsequent distraction due to the presence of the worms, or indirectly, via iron-deficiency and malnutrition [3, 71, 72]. However, *Schistosoma* infection or non-treatment was not associated with performance in tests of innate intelligence or reaction time. Inferences based on most pooled estimates for psychometrically assessed endpoints were generally insensitive to study design, S*chistosoma* species, and risk of study bias. However, associations between infection and educational outcomes were sensitive to study design and study quality–especially estimates for impact on scholastic achievement. The association between infection and scholastic achievement was directionally similar and statistically robust regardless of *Schistosoma* species; however, average effects were substantially larger for *S*. *haematobium* compared to *S*. *mansoni* infection. Cohen’s criteria for effect size suggest that the average *Schistosoma* infection-related deficits in education, learning, and memory performance range from ‘small’ to ‘moderate’. *S*. *haematobium* infection was associated with relatively larger deficits in scholastic achievement.

Prior meta-analysis of four randomized controlled trials that evaluated cognitive impacts of STH infections–which sometimes co-occur with schistosomiasis, have reached a different conclusion about the cognitive and scholastic effects of STH infection and the impact of interval treatments for STH [25, 26]. Those reviews concluded that there was substantial evidence that deworming for soil-transmitted helminth infections does not yield a cognitive or educational benefit. We note that our approach differed from these prior STH-based meta-analyses on several grounds: a) we included both interventional and observational studies to take advantage of all research data available on this question, b) we evaluated *Schistosoma-*associated impacts on two domains of educational loss (attendance and achievement), c) we further defined domains of cognitive function based on psychometrically-assessed testing to include the following: learning, memory, attention, and intelligence and d) the intervention, as defined in this meta-analysis, denoted treatment for *Schistosoma* infection, whether or not the study was randomized.

We intentionally included data from all available epidemiologic studies–whether interventional or observational in design, in this first systematic review and meta-analysis of *Schistosoma* infection-related differences in cognitive and educational outcomes. The inclusion of all available evidence reflects current standards for clinical evidence-gathering to inform health policy, in order to shape clinical practice based on the ‘best available’ relevant information [23, 24]. Future randomized-controlled trials to address this question are expected to be limited in scope and may be considered ethically objectionable given the current widespread adoption of deworming for schistosomiasis and STH. The current adoption of ‘preventive chemotherapy’ guidelines has been based on helminth-associated adverse effects on anemia and child growth. In consequence, meta-analysis of available evidence, as performed in the present study, remains the most practical strategy to inform current policy. By this approach, we have identified ‘small to moderate’ infection-related deficits in education, learning, and memory performance using the Cohen’s criteria of effect size. However, such numerically small deficits (per Cohen’s criteria) may significantly underestimate clinical significance of infection for childhood development, as *Schistosoma* infection is an exposure affecting millions of children in endemic regions. Hence, small to moderate deficits at the individual level may amount to large and important differences in disease burden at the population level [73, 74]. Our interpretation of SMD estimates is ultimately grounded in the importance of summary measures for clarifying existing knowledge gaps regarding the relationship of *Schistosoma* infections to respective outcomes, and the expected benefit of systematically lowering infection-related deficits in the millions of children at risk.

As a limitation of our approach, we acknowledge the possibility of residual confounding and bias in the primary literature, given that majority of included studies were observational or non-randomized intervention trials. Only two of the 30 included studies used an RCT design, thus a sensitivity analysis based on RCT vs. non-RCT study design was not possible. However, as part of sensitivity analysis we evaluated the potential for differences in pooled estimates based on our expanded definition of intervention as including longitudinal studies that included praziquantel treatment. Investigation of pooled estimate sensitivity by region of study, the year of publication, and by risk of bias did not result in materially different statistical inferences. Specific investigation of publication bias suggests that any possible greater likelihood of publishing positive studies did not unduly influence observed findings. Our approach of including both interventional and non-interventional studies is consistent with theoretical and empirical evidence that meta-analyses based on observational studies generally produce estimates of effect similar to those from meta-analyses based on randomized controlled trials, and that *a priori* exclusion of observational studies in systematic reviews is inappropriate and inconsistent with the evidence-based medical decision-making approach [75, 76]. In addition, the often restrictive inclusion criteria and short follow-up duration in RCTs could easily result in outcomes largely different from when the same interventions are applied to a general population.

Despite the intuitive appeal of our cognitive domain based evaluation, we acknowledge the critique that our classification of psychometric instruments by domain required some level of subjectivity, especially for tools that capture performance across multiple domains. We explicitly identified the instruments used in each study and, based on literature description of the major cognitive domain assessed by each tool, combined related tools into four separate domains. Ultimately, each instrument was assigned to one cognitive domain only. We have described the logic for our choices in the supplementary information (**S1 Table**) to provide a basis for further discussion and give sufficient context for critical evaluation of our approach in developing future studies.

Our decision to combine psychometric evaluations of cognitive functions in four domains (based on the primary capacity being tested) is a strength of our empirical approach. By so doing, we recognize that *Schistosoma* infection may not have equal impact on all cognitive domains. For example, to the extent that innate intelligence is strongly influenced by fixed or heritable factors, we did not anticipate infection related differences on tests of ‘intelligence quotient’. Unlike intelligence tests, we considered the other tests of memory, learning, and reaction time to be more sensitive to infection, and thus modifiable by presence/non-treatment vs. absence/treatment of *Schistosoma* infection. Unexpectedly, reaction time was not associated with *Schistosoma* infections. However, our findings of infection-related reductions in learning and memory tests are consistent with our hypotheses that these cognitive domains are sensitive to adverse environmental perturbations–including *Schistosoma* infection.

### Remaining gaps, and recommendations for future research

The finding of infection-related cognitive deficits and educational loss reported here is clinically and health policy-relevant for mitigating the cognitive and functional morbidities of *Schistosoma* spp. infection in children. The ‘small-to-moderate’ effects demonstrated may, in reality, be an underestimate of the lifetime impact of *Schistosoma* infection on personal performance (as affected via ultimately irreversible educational and cognitive losses). Typical epidemiologic studies necessarily include only a constrained portion of the relevant etiologic period. Among school-age children, *Schistosoma* infection is often effectively already chronic and/or recurrent, with reinfection rates extremely high in the absence of meaningful environmental interventions to reduce re-infection following treatment. The cumulative cognitive and educational impact of persistent infection may not be adequately captured by the relatively short-term investigations of treatment impact included in this meta-analysis.

In schistosomiasis*-*endemic regions, many children are infected by age two and remain chronically infected through school-age and late adolescence [9]. Under current national *Schistosoma* control treatment guidelines, preschool-age children are not treated as part of routine deworming programs for STH or schistosomiasis [1, 6, 30]. These children may therefore suffer cumulative damage to their health and function that is currently not reflected in most short-term study outcomes (or this meta-analysis). Of note, recent investigations have demonstrated the safety and efficacy of praziquantel for treatment of *Schistosoma* infection in preschool children [9]. The existence of an adverse developmental impact of *Schistosoma* infection on cognitive/educational domains would be a major justification for expanding the age-bracket of children who should be treated with praziquantel. Currently, evidence suggests that the timing of infection across a life path is especially consequential in terms of the severity of cognitive and physiologic impairments experienced [77, 78]. Future investigations evaluating the relative differences in cognitive outcomes for pre-school children with and without *Schistosoma* infection will be important for understanding the magnitude of potential impact of better prevention of *Schistosoma* infection.

It is currently unknown whether the cognitive and educational loss associated with *Schistosoma* infection can be reversed with treatment alone. The fact that infection often occurs in the context of malnutrition, coincident parasitic infections, and extreme poverty suggests that cognitive remediation efforts will need to be multi-faceted, using an integrated disease management framework. We expect that educational and cognitive interventions will be most effective if initiated earlier in life and that the package of interventions may need to include remedial instruction, the prevention of reinfection for treated/cured children, management of comorbid health conditions, and interventions for improvement of nutritional status.

### Policy implications

Our investigation suggests that *Schistosoma* infection/non-treatment is associated with educational and cognitive loss. Our findings further suggest a definite cognitive and educational benefit of anti-schistosomal deworming among school-age children. Future complex intervention studies of early childhood interventions, focused on improving child well-being and cognitive potential, are needed to determine to what extent these observed deficits are preventable or reversible. Interventions that employ an integrated disease management framework will likely identify cost-efficiencies for leveraging existing disease and nutrition treatment programs in helminth-affected regions.

## Supporting information

S1 Text PROSPERO protocol CRD42016040052 registered for this study. (PDF)

S2 Text Schematic of search strategy for the systematic review. (DOCX)

S3 Text Summary of supplemental data. (DOCX)

S1 Table Classification of individual study assessments into domains based on psychometric evaluation of cognitive function & education-related assessments. (DOCX)

S2 Table The impact of *Schistosoma* infection/non-treatment on educational loss, memory, and learning domains with serial exclusion of individual studies–a sensitivity analysis quantifying the influence of individual studies. (DOCX)

S3 Table PRISMA checklist. (DOCX)

S1 Fig Sensitivity analysis of the impact of publication year on pooled estimates for educational and cognitive loss domains. (DOCX)
