# Supplementary material for: Cognitive deficits and educational loss in children with schistosome infection—A systematic review and meta-analysis
Source: PLoS Negl Trop Dis. 2018 Jan 12;12(1):e0005524. doi: 10.1371/journal.pntd.0005524 (PMC5766129; doi:10.1371/journal.pntd.0005524)
Supplement: S1 Text — (PDF) [file pntd.0005524.s001.pdf]

PROSPERO International prospective register of systematic reviews

Cognitive development and loss of education with human Schistosoma species infection: a systematic review and meta-analysis

Amara Ezeamama, Amaya Bustinduy, Allan Nkwata, Leonardo Martinez, Noel Pabalan, Michael Boivin, Charles King

Citation

Amara Ezeamama, Amaya Bustinduy, Allan Nkwata, Leonardo Martinez, Noel Pabalan, Michael Boivin, Charles King. Cognitive development and loss of education with human Schistosoma species infection: a systematic review and meta-analysis. PROSPERO 2016:CRD42016040052 Available from http://www.crd.york.ac.uk/PROSPERO_REBRANDING/display_record.asp?ID=CRD42016040052

Review question(s)

Is infection with Schistosoma in children or failure to treat infection associated with childhood educational loss and cognitive deficits?

Searches

Electronic databases including MEDLINE, Web of Science, and BIOSIS Previews were searched for original research articles, conference abstracts, and dissertations.

Search terms included variations and combinations of the following keywords: “bilharzia”, “schistosomiasis”, “Schistosoma”, “school attendance”, “attention”, “impairment”, “memory”, “cognition”, amongst others.

Studies were included if they evaluated cognitive function in children using any psychometric tests or measured school attendance or achievement in relation to infection by Schistosoma parasites of any species.

No restrictions were made according to language, design or publication date.

Experts with research interests in schistosomiasis-associated morbidities were consulted for additional listings.

Types of study to be included

No restrictions on the study design were made. Longitudinal and cross-sectional/case-control studies were included. This is because we wanted to pool together all the available literature on this question.

Condition or domain being studied

Schistosomiasis is a preventable disease caused by infection with blood flukes of the genus Schistosoma. It is transmitted when humans come into contact with fresh water contaminated with parasites released from infected fresh water snails, the intermediate hosts. An estimated 800 million persons in tropical and sub-tropical countries are at risk of infection by one of three main human Schistosoma species parasites – S. mansoni, S. haematobium, and S. japonicum. As many as 240 million adults and children are actively infected, the majority living in Sub-Saharan Africa. Children are often infected by two years of age and many remain chronically infected throughout their school-age years. Despite the fact that periodic mass drug administration with praziquantel is recommended in these settings, schistosoma infected children are not routinely treated, partly because of a lack of safe pediatric doses.

Participants/ population

Studies were included if they evaluated cognitive function in school-going children between the ages of 5 to 19 years or measured school attendance or achievement in relation to infection by Schistosoma parasites.

Studies were excluded if they reported no outcome measure of interest to the investigators, were not original articles, were review articles, or provided no infection measures.

Intervention(s), exposure(s)

Epidemiologic studies that assessed differences in cognitive test scores (based on psychometric tests) and/or educational status (measured as scholastic achievement and school attendance rate) in relation to Schistosoma infection or treatment were included in the study.

No restrictions based on study design, language or publication date were made.

Comparator(s)/ control

Cognitive and educational deficits in untreated children with schistosomiasis compared with those that received treatment during the same period.

Context

Epidemiologic studies have associated Schistosoma infections with adverse impacts on anemia, growth, fitness, pediatric quality-of-life, and sub-optimal child development.

Outcome(s)

Primary outcomes

Psychometrically assessed cognitive function in four domains: memory, learning/executive function, attention/reaction time and intelligence.

Secondary outcomes

Educational loss measured as school attendance and scholastic achievement.

Data extraction, (selection and coding)

Data extraction was performed by two trained researchers who independently screened identified unique articles by title and abstract. Disagreements between reviewers were resolved by consensus usually involving a third reviewer.

Risk of bias (quality) assessment

We followed PRISMA guidelines as well as checklists of Meta-analysis of Observational Studies in Epidemiology (MOOSE) for observational studies. Quality assessment of observational studies included is being implemented using the modified center for evidence based medicine rating scheme and the modified Ottawa scale.

Strategy for data synthesis

Aggregate data from each study were used with the aim of deriving Standardized Mean Difference (SMD) estimates and 95% confidence intervals for each measure of cognitive or school based function.

Analysis of subgroups or subsets

Sub-group analyses where conducted to explain heterogeneity between studies, if found to be unacceptably high. Where possible, meta-regression was conducted to further explain heterogeneity.

Dissemination plans

Presentation to the SCORE meetings and to the American Society of Tropical Medicine and Hygiene National Meeting 2014. Publication in a peer-reviewed journal.

Contact details for further information

Allan Nkwata

101 Buck Road

Athens GA, 30601

kizzalan@uga.edu

Organisational affiliation of the review

None

Review team

Dr Amara Ezeamama, Department of Psychiatry, College of Osteopathic Medicine, Michigan State University
Dr Amaya Bustinduy, Department of Clinical Research, London School of Hygiene and Tropical Medicine, London, UK
Mr Allan Nkwata, Department of Epidemiology and Biostatistics, College of Public Health, University of Georgia
Mr Leonardo Martinez, Department of Epidemiology and Biostatistics, College of Public Health, University of Georgia
Dr Noel Pabalan, Center for Research and Development, Angeles University Foundation, Angeles City, The Philippines
Dr Michael Boivin, Department of Psychiatry, College of Osteopathic Medicine, Michigan State University
Dr Charles King, Center for Global Health and Diseases, Case Western Reserve University School of Medicine, Cleveland, Ohio, USA

Anticipated or actual start date

10 July 2014

Anticipated completion date

31 October 2016

Funding sources/sponsors

Schistosomiasis Consortium for Operational Research and Evaluation (SCORE). University of Georgia, Athens, GA, USA

Conflicts of interest

None known

Language

English

Country

England, Philippines, United States of America

Subject index terms status

Subject indexing assigned by CRD

Subject index terms

Adolescent; Child; Child Development; Cognition; Cognition Disorders; Education; Educational Status; Humans; Learning; Schistosoma; Schools; Students

Stage of review

Completed but not published

Date of registration in PROSPERO

27 June 2016

Date of publication of this revision

07 September 2016

Stage of review at time of this submission	Started	Completed
Preliminary searches	Yes	Yes
Piloting of the study selection process	Yes	Yes
Formal screening of search results against eligibility criteria	Yes	Yes
Data extraction	Yes	Yes
Risk of bias (quality) assessment	Yes	Yes

Data analysis	Yes	Yes

PROSPERO
International prospective register of systematic reviews
The information in this record has been provided by the named contact for this review. CRD has accepted this information in good faith and registered the review in PROSPERO. CRD bears no responsibility or liability for the content of this registration record, any associated files or external websites.
