# Supplementary material for: Cognitive deficits and educational loss in children with schistosome infection—A systematic review and meta-analysis
Source: PLoS Negl Trop Dis. 2018 Jan 12;12(1):e0005524. doi: 10.1371/journal.pntd.0005524 (PMC5766129; doi:10.1371/journal.pntd.0005524)
Supplement: S2 Text — (DOCX) [file pntd.0005524.s002.docx]

**Computer-assisted online search strategy**

**Pubmed**

Search used:

("schistosomiasis"[ MeSH Terms ] OR *Schistosoma* [MeSH] OR schisto*[tiab])

AND

("school attendance"[tiab] OR "attainment*"[All Fields] OR “cognit*”[WORD] OR "impairment"[All Fields] OR "educ*"[TI] OR "memory"[MeSH] OR "learning"[WORD] OR“attention” [All Fields])

Results: 409 hits

**Biosis**

Search used:

("schistosomiasis"[TOPIC] OR "schistosomiasis"[TOPIC] OR schisto*[TITLE])

AND

("school attendance"[TOPIC] OR "attainment*"[TOPIC] OR cognition [TOPIC] OR cognition [TITLE] OR "impairment"[TOPIC] OR "impairment"[TITLE] OR "child"[TOPIC] OR "learning"[TOPIC] OR“attention” [TOPIC])

Results: 1997 hits

**Web of Science**

Search used:

[("schistosomiasis"[TOPIC] OR "schistosomiasis"[TOPIC] OR schisto*[TITLE])

AND

("school attendance"[TOPIC] OR "attainment*"[TOPIC] OR cognition [TOPIC] OR cognition [TITLE] OR "impairment"[TOPIC] OR "impairment"[TITLE] OR "child"[TOPIC] OR "learning"[TOPIC] OR“attention” [TOPIC])

Results: 1588 hits
