# Supplementary material for: Cognitive deficits and educational loss in children with schistosome infection—A systematic review and meta-analysis
Source: PLoS Negl Trop Dis. 2018 Jan 12;12(1):e0005524. doi: 10.1371/journal.pntd.0005524 (PMC5766129; doi:10.1371/journal.pntd.0005524)
Supplement: S3 Text — (DOCX) [file pntd.0005524.s003.docx]

**Summary of Supplemental Data**

Our search strategy is detailed in “S2 Text online search strategy”. Our rationale for classification of cognitive and educational domains are demonstrated in Table S1. The direction and magnitude of the pooled estimate for *Schistosoma* infection/non-treatment-related adverse impacts on both domains of educational loss was robust to deletion of all studies (Table S2). Similarly, the pooled estimate of *Schistosoma* infection impact on memory was robust to deletion of all but Jukes, et al. (SMD with omission = -0.23 (95% CI: -0.50, 0.03) and Nazel, et al., (SMD with omission = -0.18 (95% CI: -0.37, 0.01) (Table S2).

Over time, the mean pooled estimates of adverse effect of *Schistosoma* infection on educational loss varied. These estimates became statistically robust by the year 1990 and remained stable thereafter (Figure S1). In contrast, the precision of infection effect on memory and learning outcomes tended to vary. Specifically, an estimated memory deficit of -0.44 (95% CI: -1.06, 0.17) was noted for the earliest study in 1974. This pooled estimate moderated to -0.13 (-0.35 to 0.09) in 1999, when only three studies had been published, and subsequently strengthened to -0.33 (95%CI: -0.60, -0.06) by 2014, when a total of eight studies were available (Figure S1).
