# Supplementary material for: Cognitive deficits and educational loss in children with schistosome infection—A systematic review and meta-analysis
Source: PLoS Negl Trop Dis. 2018 Jan 12;12(1):e0005524. doi: 10.1371/journal.pntd.0005524 (PMC5766129; doi:10.1371/journal.pntd.0005524)
Supplement: S1 Table — (DOCX) [file pntd.0005524.s004.docx]

**Table S1-Classification of Psychometric Evaluations & Education-Related Assessments into Domains**

| ***Cognitive Domains*** | ***Psychometric Tests Combined*** | ***Description of Dominant Cognitive Construct Captured by Respective Instruments*** |
| --- | --- | --- |
| ***Memory*** | Corsi Block | Test of visual working memory^1^ |
|  | Verbal Fluency (measures LT memory) | Speed of scanning and retrieval from memory. Measures long-term semantic memory^2,3^ |
|  | WISC-digit span, Digit Span – forwards/backwards | Working memory phonological^2,3^ |
|  | Wide-range assessment of memory and learning (memory domain) – i.e. WRAML Memory | A measure of verbal and working memory in children^4,5^ |
|  | Free Recall | Measure of long-term auditory memory^1^ |
|  | Weschler Memory Scale | Designed to measure different memory functions in children |
| ***Reaction Time***  ***and***  ***Attention*** | Choice Reaction Time | Speed of processing^2^ |
|  | Grooved Peg Board | Psychomotor skills and manual dexterity^2,3^ |
|  | Test of everyday attention for children (TEA-Ch) | A measure of sustained attention^6^ |
|  | Picture search | A measure of focused attention. Related to the executive system of working memory^1^ |
|  | Silly Sentences | Speed of verbal processing^2^ |
|  | Continuous Performance Tests | A measure of attention related problems in children and the capacity of children to maintain vigilance. |
|  | Digit Cancellation Tests | Designed to measure selective attention deficits in children |
|  | Trail Making Test | Measure of visual attention and task switching |
|  | WISC-coding | Assesses children's abilities to focus attention and quickly scan, discriminate between, and sequentially order visual information. Speed of Information Processing |
|  | Coding/Code transmission Tests | Assesses children's abilities to focus attention and quickly scan, discriminate between, and sequentially order visual information. Speed of Information Processing |
| ***Learning and***  ***Executive Function*** | Wide-range assessment of memory and learning (learning domain) – i.e. WRAML Learning | Assesses a child’s ability to learn and recall new information |
|  | Spanish Vocabulary Learning | Assessing the mastery/learning of Spanish names for objects presented to children equally unfamiliar with the Spanish language^2^ |
|  | Stroop (All kinds of this test) | Executive function/ speed of processing^2^ |
|  | Verbal analogies |  |
|  | Wisconsin Card Sorting Test | A measure of cognitive flexibility in children – i.e. capacity to be flexible given changing schedules.  Used primarily to assess cognitive flexibility and abstract thinking. |
|  | Reciprocal Motor Programs Test | Used to assess a component of executive function – i.e. the capacity of children to shift between tasks. |
|  | Comprehension - Assessed in Nokes 1992^7^ |  |
|  | Matching Familiar Figures Test |  |
|  | Rey Auditory Verbal Learning Test |  |
|  | WISC-III Arithmetic |  |
|  | Oddity Learning |  |
| ***IQ based Tests*** | Philippine non-verbal intelligence test (PNIT) | Assessment of non-verbal fluid intelligence. |
|  | WISC (if specific sub-scales are not named) | Assesses a child’s intellectual performance. Measures intellectual performance as a multidimensional construct |
|  | Raven's progressive matrix | An assessment of general intelligence |
|  | Peabody Picture Vocabulary Test - General Intelligence | Assesses a child’s intellectual performance. Measures intellectual performance as a multidimensional construct |
|  | Kaufmann Assessment Battery for Children - (Total mental processing + nonverbal total) | Assesses intelligence, reasoning and achievement in children |
|  |  |  |
| ***School Attendance*** | Enrollment rate | Percent of children in a given locale enrolled in school |
|  | School performance | Tests of scholastic achievement were generally not psychometric instrument based. They included a range of assessments typically administered by teachers or researchers for the purpose of ranking pupil performance. |
|  | Absenteeism rate | Number of days a child who is enrolled in school misses school within specified period of time. |
|  |  |  |
| ***Achievement*** | Arithmetic | Tests of scholastic achievement were generally not based on psychometric instrument testing. They included a range of assessments typically administered by teachers or researchers for the purpose of ranking pupil performance. |
|  |  |  |
|  | Reading /vocabulary |  |
|  | Spelling |  |
|  | Scholastic achievement |  |
|  | General education Tamil Test |  |
|  | General education Math Test |  |
|  | Arithmetic Test |  |
|  |  |  |
| ***Other cognitive measures in Castle 1974 study and how they were classified*** | **Description per Study Authors** | **Final Classification for Meta-analysis** |
| Verbal meaning | Tests the ability to understand ideas expressed in words | Learning |
| Reasoning | Tests the ability to solve logical problems | Learning |
| Space | Tests the ability to visualize and mentally manipulate objects in two or three dimensions | Memory |
| Number | Tests ability to work with figures and mentally manipulate objects in two or three dimensions | Learning |
| Word Fluency | To test vocabulary | Memory |
| Scholastic Ability | Combination of verbal meaning & verbal fluency | Scholastic Achievement |
|  |  |  |

**References**

1. Nokes C, McGarvey ST, Shiue L, et al. Evidence for an improvement in cognitive function following treatment of *Schistosoma japonicum* infection in Chinese primary schoolchildren. *Am J Trop Med Hyg* 1999; **60**(4): 556-65.

2. Jukes MC, Nokes CA, Alcock KJ, et al. Heavy schistosomiasis associated with poor short-term memory and slower reaction times in Tanzanian schoolchildren. *Trop Med Int Health* 2002; **7**(2): 104-17.

3. Sakti H, Nokes C, Hertanto WS, et al. Evidence for an association between hookworm infection and cognitive function in Indonesian school children. *Trop Med Int Health* 1999; **4**(5): 322-34.

4. Ezeamama AE, Friedman JF, Acosta LP, et al. Helminth infection and cognitive impairment among Filipino children. *Am J Trop Med Hyg* 2005; **72**(5): 540-8.

5. Ezeamama AE, McGarvey ST, Hogan J, et al. Treatment for *Schistosoma japonicum*, reduction of intestinal parasite load, and cognitive test score improvements in school-aged children. *Plos Neglect Trop Dis* 2012; **6**(5).

6. Hurlimann E, Houngbedji CA, N'Dri PB, et al. Effect of deworming on school-aged children's physical fitness, cognition and clinical parameters in a malaria-helminth co-endemic area of Cote d'Ivoire. *BMC Infect Dis* 2014; **14**: 411.

7. Nokes C, Grantham-McGregor SM, Sawyer AW, Cooper ES, Robinson BA, Bundy DA. Moderate to heavy infections of *Trichuris trichiura* affect cognitive function in Jamaican school children. *Parasitology* 1992; **104 ( Pt 3)**: 539-47.
