# Supplementary material for: Cognitive deficits and educational loss in children with schistosome infection—A systematic review and meta-analysis
Source: PLoS Negl Trop Dis. 2018 Jan 12;12(1):e0005524. doi: 10.1371/journal.pntd.0005524 (PMC5766129; doi:10.1371/journal.pntd.0005524)
Supplement: S2 Table — (DOCX) [file pntd.0005524.s005.docx]

Table S2: The Impact of *Schistosoma* Infection/non-treatment on educational loss, memory, and learning domains with serial deletion of individual studies – Quantifying influence of individual studies

| Study Omitted | Pooled estimate with deletion | 95% Confidence Interval | | Impact of Deletion on Pooled Estimate |
| --- | --- | --- | --- | --- |
| 1. Attendance |  |  |  |  |
| Epstein (1974) | -0.38 | -0.63 | -0.13 | Robust |
| Ejezie (1981) | -0.26 | -0.48 | -0.03 | Robust |
| El-Hawy (1990) | -0.29 | -0.52 | -0.06 | Robust |
| Ekanem (1994) | -0.33 | -0.58 | -0.09 | Robust |
| Hussien-L. Egypt (1996) | -0.36 | -0.68 | -0.06 | Robust |
| Hussien-U. Egypt (1996) | -0.35 | -0.66 | -0.04 | Robust |
| Clerq et al (1998) | -0.37 | -0.62 | -0.13 | Robust |
| Nazel (1999) | -0.38 | -0.62 | -0.13 | Robust |
| Useh & Ejezie (1999) | -0.36 | -0.61 | -0.11 | Robust |
| Beasley (2000) | -0.39 | -0.64 | -0.14 | Robust |
| Meremikwu (2000) | -0.41 | -0.65 | -0.17 | Robust |
| Fentiman & Hall (2001) | -0.36 | -0.60 | -0.11 | Robust |
| Tiruneh (2001) | -0.46 | -0.65 | -0.26 | Robust |
| Miguel (2004) | -0.36 | -0.61 | -0.11 | Robust |
| Mehkimer (2005) | -0.35 | -0.59 | -0.10 | Robust |
| Rasoamanamihaja (2016) | -0.36 | -0.62 | -0.10 | Robust |
| Combined Attendance | -0.36 | -0.60 | -0.12 |  |
|  |  |  |  |  |
| 1. Achievement |  |  |  |  |
| Loveridge (1948) | -0.46 | -0.82 | -0.10 | Robust |
| Jordan (1962) | -0.55 | -0.94 | -0.16 | Robust |
| Goldin (1972) | -0.63 | -1.02 | -0.24 | Robust |
| Castle (1974) | -0.61 | -1.00 | -0.22 | Robust |
| Epstein (1974) | -0.61 | -1.01 | -0.22 | Robust |
| Ejezie (1981) | -0.44 | -0.73 | -0.16 | Robust |
| Haycock (1983) | -0.59 | -1.01 | -0.17 | Robust |
| El-Hawy (1990) | -0.55 | -0.95 | -0.15 | Robust |
| Ekanem (1994) | -0.63 | -1.02 | -0.24 | Robust |
| Clercq (1998) | -0.57 | -0.97 | -0.17 | Robust |
| Nazel (1999) | -0.59 | -0.98 | -0.19 | Robust |
| Meremikwu (2000) | -0.59 | -1.00 | -0.18 | Robust |
| Jukes (2002) | -0.62 | -1.02 | -0.22 | Robust |
| Miguel (2004 | -0.61 | -1.01 | -0.21 | Robust |
| Grigorenko (2006) | -0.63 | -1.02 | -0.24 | Robust |
| Terer (2013) | -0.62 | -1.02 | -0.21 | Robust |
| Combined Achievement | -0.58 | -0.96 | -0.20 |  |
|  |  |  |  |  |
| 1. Memory |  |  |  |  |
| Grigorenko (2006) | -0.27 | -0.54 | 0.00 | Robust |
| Ezeamama (2012) | -0.30 | -0.58 | -0.01 | Robust |
| Castle (1974) | -0.32 | -0.60 | -0.04 | Robust |
| Ezeamama (2005) | -0.30 | -0.59 | 0.00 | Robust |
| Nazel (1974) | -0.18 | -0.37 | 0.01 | Robust |
| Jukes (2002) | -0.23 | -0.50 | 0.03 | Robust |
| Hurlimann (2014) | -0.33 | -0.61 | -0.05 | Robust |
| Nokes (1999) | -0.33 | -0.61 | -0.06 | Robust |
| Combined Memory | -0.28 | -0.53 | -0.03 |  |
|  |  |  |  |  |
| 1. Learning |  |  |  |  |
| Castle (1974) | -0.41 | -0.81 | -0.02 | Robust |
| Epstein (1974) | -0.27 | -0.48 | -0.06 | Robust |
| Jukes (2002) | -0.35 | -0.73 | -0.02 | Robust |
| Ezeamama (2005) | -0.48 | -0.80 | -0.15 | Robust |
| Grigorenko (2006) | -0.41 | -0.75 | -0.06 | Robust |
| Ezeamama (2012) | -0.45 | -0.80 | -0.11 | Robust |
| Combined Learning | -0.40 | -0.70 | -0.09 |  |
|  |  |  |  |  |

Note: An influential study is one whose exclusion results in a change in direction of the pooled estimate.
