# Supplementary material for: Cognitive deficits and educational loss in children with schistosome infection—A systematic review and meta-analysis
Source: PLoS Negl Trop Dis. 2018 Jan 12;12(1):e0005524. doi: 10.1371/journal.pntd.0005524 (PMC5766129; doi:10.1371/journal.pntd.0005524)
Supplement: S1 Fig — (DOCX) [file pntd.0005524.s007.docx]

Figure S1: Sensitivity analysis of the impact of publication year on pooled estimates for educational and cognitive loss domains
